# Rehabilitation applications and progress in managing pulmonary hypertension

**DOI:** 10.3389/fmed.2025.1659818

**Published:** 2025-08-14

**Authors:** Yuyan Liu, Xiao Guo, Panpan Liu, Shuying Jia, Fang Wang, Fei Li, Ping Yuan, Xuejing Wang

**Affiliations:** ^1^Rehabilitation Medicine College, Shandong Second Medical University, Weifang, China; ^2^Department of Cardio-Pulmonary Circulation, Innovation and Incubation Center (IIC), Shanghai Pulmonary Hospital, School of Medicine, Tongji University, Shanghai, China; ^3^Bioenvironmental Science Program, Morgan State University, Baltimore, MD, United States; ^4^Department of Radiology, Shanghai Pulmonary Hospital, School of Medicine, Tongji University, Shanghai, China

**Keywords:** pulmonary hypertension, rehabilitation, exercise limitation, exercise, quality of life

## Abstract

Pulmonary hypertension (PH) is a significant global health challenge that profoundly impacts exercise tolerance and quality of life for affected individuals. While advancements in pharmacological therapies have improved symptom management, exercise capacity, and overall well-being, there is a growing recognition of rehabilitation therapy as a promising non-pharmacological approach. Rehabilitation therapy aims to restore or enhance physical, psychological, and social functioning in individuals compromised by disease, injury, or congenital conditions through an integrated approach encompassing medical, social, and educational interventions. In the context of PH, rehabilitation therapy encompasses diverse modalities, including exercise training, inspiratory muscle training, neuromuscular electrical stimulation, and psychological support. These interventions target key symptoms such as exercise intolerance and dyspnea, with the goal of improving patient outcomes and fostering a return to independent and meaningful living. Emerging evidence demonstrates that rehabilitation therapy is both safe and effective in enhancing cardiopulmonary function, muscle strength, and exercise capacity. Furthermore, it has been shown to alleviate anxiety and depression, improve quality of life, and facilitate reintegration into familial and social roles. This review provides a comprehensive overview of the clinical features, prevalence, and current treatment landscape of PH, with a particular focus on the role and advancements in rehabilitation therapy. We discuss the therapeutic potential of rehabilitation therapy in addressing the multifaceted challenges of PH and explore future directions and innovative approaches in this evolving field. By highlighting the clinical utility and research progress of rehabilitation therapy, this review aims to offer new insights and perspectives for optimizing the management of PH and improving patient outcomes.

## 1 Introduction

PH is a complex and progressively worsening cardiopulmonary disease affecting approximately 1% of the global population, posing a significant health burden on patients (1). Despite substantial advancements in pharmacologic therapies, the majority of patients continue to suffer from varying degrees of right heart dysfunction (2). As a result, they often experience reduced exercise capacity and a markedly diminished quality of life (3, 4). Currently, despite the availability of numerous targeted therapies, PH remains an incurable condition (5). Contrary to earlier beliefs, impaired exercise tolerance in PH is not solely attributable to central cardiopulmonary dysfunction. Recent studies have demonstrated that peripheral factors—particularly skeletal muscle and cerebrovascular impairments—also contribute significantly to exercise limitation (6–9). The pathophysiological mechanisms underlying skeletal muscle dysfunction in PH patients can be broadly categorized into three key domains: mitochondrial abnormalities, microcirculatory disturbances, and changes in muscle morphology (10).

In PH patients, mitochondrial structure and density within skeletal muscle are significantly reduced, accompanied by downregulation of critical proteins in the electron transport chain. This results in impaired mitochondrial metabolism and reduced cellular energy production. A hallmark of skeletal muscle dysfunction in PH is diminished systemic oxygen uptake. Lower oxygen saturation levels and decreased capillary density further compromise oxygen delivery and utilization. Additionally, reduced expression of microRNA-126 (miR-126), which plays a role in microvascular development and maintenance, exacerbates capillary rarefaction and impairs muscle oxygenation. Furthermore, the maximum contractile force and active stiffness of type II muscle fibers are markedly diminished. Myosin expression is reduced, while muscle atrophy and increased signaling for protein degradation are observed. There is also a notable shift in fiber type from type I to type II muscle fibers, which further decreases muscle endurance and strength. The combined effects of mitochondrial, microvascular, and myofibrillar abnormalities lead to severe peripheral muscle dysfunction and are a major contributor to the exercise intolerance characteristic of PH.

Therefore, despite advances in drug therapy, the long-term management of PH remains challenging. Exploring novel therapeutic strategies—particularly those targeting peripheral muscle dysfunction—is crucial for improving patient outcomes. Importantly, rehabilitation therapy can improve the characteristics of peripheral muscles (11). This review aims to provide a comprehensive overview of PH, including its clinical manifestations, epidemiology, and current treatment strategies. Particular emphasis is placed on the role of rehabilitation therapy, highlighting recent advances and evidence supporting its ability to enhance mobility, muscle function, and overall quality of life in patients with PH. Furthermore, the potential of emerging rehabilitation approaches in the management of PH will be discussed. By synthesizing these aspects, this review seeks to offer deeper insights into the evolving landscape of PH treatment, with a focus on rehabilitation therapy as a promising adjunctive strategy. Ultimately, improving our understanding of these therapeutic options may not only enhance clinical outcomes and prognosis for patients with PH but also inspire new directions in the research and management of related cardiopulmonary disorders.

## 2 Fundamental concepts in pulmonary hypertension

### 2.1 Definition of pulmonary hypertension

PH is a group of life-threatening pulmonary vascular disorders characterized by vasoconstriction and remodeling, leading to elevated pulmonary vascular pressure and resistance, ultimately resulting in death from right heart failure (12). Definitive diagnosis requires invasive hemodynamic evaluation via right heart catheterization, with PH defined by a mean pulmonary artery pressure (mPAP) ≥ 20 mmHg and a pulmonary capillary wedge pressure (PCWP) ≤ 15 mmHg (12). According to the World Health Organization (WHO), PH is classified into five major groups based on etiology: (1) pulmonary arterial hypertension (PAH), (2) PH associated with left heart disease, (3) PH associated with lung diseases and/or hypoxia, (4) PH associated with pulmonary artery obstructions, and (5) PH with unclear and/or multifactorial mechanisms. The prevalence varies significantly across these groups, with PH due to left heart disease being the most common subtype. Notably, the overall prevalence of PH has been increasing in recent years (13). Despite ongoing research, the underlying pathophysiological mechanisms of PH remain incompletely understood. However, irrespective of the causative classification, all subtypes of PH share a common pathological consequence: right ventricular remodeling, which can ultimately progress to right heart failure and death (14, 15).

### 2.2 Clinical manifestations and management of pulmonary hypertension

In the early clinical stages, the symptoms of PH are often nonspecific and easily misattributed to other conditions. Therefore, the early detection of PH may be difficult solely relying on clinical manifestations. Systematic diagnostic methods are needed, including both functional assessment and the use of advanced imaging techniques. The initial assessment typically begins with a comprehensive analysis of clinical manifestations, such as exertional dyspnea, fatigue, and syncope. Electrocardiogram (ECG) examinations may reveal the load pattern of the right ventricle, while chest X-rays can show structural changes in the heart and lungs. Through pulmonary function tests combined with arterial blood gas analysis, key information regarding ventilation efficiency and abnormal gas exchange can be provided. For a clear assessment, echocardiography serves as the main screening tool for estimating pulmonary artery pressure and evaluating right heart function. Ventilation/perfusion imaging remains indispensable for detecting the cause of thromboembolic events. The final diagnosis is based on right heart catheterization. As the disease progresses, symptoms are increasingly provoked by minimal physical activity and manifest at rest. Patients may develop chest pain, exertional syncope, lower extremity edema, and hepatic congestion—classic signs of right heart failure—indicating disease progression (12, 16). Overall, the clinical manifestations of PH range from mild to severe and evolve gradually, closely associated with the development of right heart failure. Beyond hemodynamic disturbances—such as elevated pulmonary vascular resistance, reduced stroke volume, and diminished cardiac index—PH also induces a range of systemic pathophysiologic changes. These include reduced skeletal muscle strength and endurance, as well as weakened respiratory muscles, particularly the diaphragm. Such impairments significantly reduce peak oxygen uptake, contributing to worsening fatigue, dyspnea, and overall functional decline. These cascading effects ultimately lead to profound exercise intolerance and markedly diminished quality of life (17).

These mechanisms highlight that PH is not merely a cardiopulmonary disorder but a systemic disease characterized by widespread metabolic and functional impairments, affecting patients’ physiological resilience and exercise tolerance through multiple pathways. Epidemiological data suggest that the incidence of PH ranges from 2.0 to 7.6 cases per million adults annually, with a prevalence between 11 and 26 cases per million. PH can affect individuals of all ages, though it is four times more common in women than in men. Interestingly, while more women are affected, men with PH tend to have poorer survival outcomes (18, 19). Current treatment strategies for PH are broadly divided into general measures, supportive therapies, and targeted pharmacologic interventions. Research has confirmed that the 14 core signaling pathways exhibit highly consistent and synergistic activation characteristics in patients with various types of PH (20). After the approval of PH-specific drugs, the 5-year survival rate of PH patients increased from 34 to 60% (21), with notable advances in symptom control, exercise capacity, hemodynamic parameters, and overall outcomes (22). However, while multi-targeted therapy shows great potential, it also faces practical challenges such as complex drug interactions, high treatment costs, and significant individual response variations.

Because PH is often diagnosed at an advanced stage, many patients already present with significant physical limitations at the time of diagnosis. Therefore, identifying additional therapeutic approaches is critical for improving prognosis and long-term functional outcomes. In this context, rehabilitation therapy has attracted increasing attention due to its low cost, ease of implementation, and noninvasive nature. Emerging evidence supports the safety and clinical efficacy of rehabilitation therapy in PH, positioning it as a promising adjunctive treatment. This approach offers new hope for enhancing the quality of life in patients living with this debilitating condition.

## 3 Strategies for implementing rehabilitation therapy

Rehabilitation therapy is increasingly recognized as a powerful, non-pharmacological strategy that not only restores or enhances physical, psychological, and social functioning in individuals affected by disease, injury, or congenital conditions, but also empowers them to reintegrate fully into family life and society. By integrating medical, social, and educational interventions, it delivers comprehensive, person-centered care that fosters optimal recovery, meaningful participation in daily activities, and a confident return to valued social roles. Rehabilitation therapy not only significantly enhances the quality of life for patients with PH, but also alleviates the associated familial and societal burdens. Moreover, it can effectively slow disease progression, address psychological challenges, and facilitate a smoother reintegration into family life and society. Therefore, the widespread promotion and application of rehabilitation concepts and techniques hold substantial value in improving the overall recovery outcomes for PH patients. We searched PubMed, EMBASE, and the Cochrane Collaboration database using the keywords “exercise training” or “rehabilitation” and “pulmonary hypertension.” In clinical practice, the application of rehabilitation therapy in PH can be broadly classified into four key areas: exercise training, inspiratory muscle training, neuromuscular electrical stimulation, and psychological intervention (Table 1). Carrying out rehabilitation therapy for patients with PH, the short-term rehabilitation goals are to rapidly alleviate symptoms such as shortness of breath and fatigue, increase the 6-minute walking distance, maintain blood oxygen saturation, and learn abdominal breathing. The long-term rehabilitation goals are to continuously improve cardiopulmonary endurance, right heart function and quality of life, reduce the frequency of acute exacerbations and hospitalizations, enable the patients to return to family, society, and work, and delay the progression of the disease to the end stage. The rehabilitation therapy team consists of occupational therapists (OT), physical therapists (PT), and speech therapists (ST). Its size is continuously expanding among medical professionals and is playing an irreplaceable core role in the medical service system. Among them, cardiopulmonary physical therapists (CPT), as sub-specialists of PT, bear the main responsibility in the rehabilitation treatment of patients with PH. Through evidence-based exercise training, breathing strategies, and lifestyle interventions, they systematically improve the patients’ cardiopulmonary endurance and quality of life.

**Table 1:** Summary of rehabilitation research on pulmonary hypertension.

Study/first author (journal) [Ref.]	Year	Type of study	Control patients (F), n	Training patients (F), n	Patients’ characteristics	Age		Training type	Duration and follow-up
						Control patients	Training patients		
da Fontoura et al. (49)	2025	RCT	14 (14)	17 (17)	PAH, CTEPH	24.67 ± 3.10	26.61 ± 4.1	RT	8 weeks in home
Stark et al. (78)	2025	RCT	35 (30)	33 (28)	PH WHO FC I–IV	51.71 ± 12.35	55.30 ± 13.53	CBT	4 weeks in home
Ozcan et al. (71)	2023	RCT	12 (11)	12 (11)	CTEPH, IPAH, CTD-PH, CHD-PH NYHA II-III	55.50 ± 19.17	49.16 ± 17.09	RT	8 weeks in home
Butāne et al. (4)	2022	RCT	10	11	PAH NYHA II-III	66	68	Exercise training + RT Strength training + Relaxation	12 weeks in home
Ertan et al. (63)	2022	RCT	12	12	IPAH, CTD-PH, CTEPH WHO FC II–III	44.25 ± 9.43	49.58 ± 9.89	Exercise training	8 weeks in hospital
Nagel et al. (37)	2021	RCT	19 (11)	24 (12)	PAH, CTEPH WHO FC II–IV	58 ± 14	55 ± 15	Exercise training	15 weeks in hospital
Naci et al. (26)	2021	RCT	16	16 (OMT), 16 (OMT + RT)	PAH WHO FC I-III	47.81 ± 12.67	47.06 ± 14.53 (OMT), 50.38 ± 9.02 (OMT + RT)	OMT or OMT + RT	8 weeks in hospital
Atef et al. (34)	2020	RCT	15	15	PAH	45–65	45–65	Exercise training	12 weeks in hospital
Kahraman et al. (47)	2020	RCT	12	12	PAH NYHA II-III	25.75–62.50	29.50–59.00	Neuromuscular electrical stimulation	8 weeks in hospital
Yılmaz et al. (73)	2020	RCT	11	12	PAH	40.18 ± 14.47	35.00 ± 13.68	Exercise training	6 weeks in hospital
Grünig et al. (55)	2020	RCT	58 (45)	58 (40)	PAH, CTEPH WHO FC II–IV	55.0 ± 12.7	52.3 ± 12.4	RT + Exercise training	15 weeks in hospital and in home
Babu et al. (75)	2019	RCT	33	34	PH	47.28 ± 15.6	51.45 ± 13.7	Exercise training	12 weeks in home
Woolstenhulme et al. (74)	2019	RCT	19 (19)	18 (18)	PAH WHO FC I-III NYHA I-III	47.9 ± 7.9	56.2 ± 8.8	Exercise training	10 weeks in hospital
Santos-Lozano et al. (15)	2019	RCT	4 (3)	5 (4)	PAH	40–56	35–53	Resistance exercise+Exercise training	8 weeks in hospital
Saglam et al. (43)	2015	RCT	17 (14)	14 (11)	PAH WHO FC II-III	52.2 ± 8.8	46.8 ± 15.6	RT	6 weeks in hospital
Weinstein et al. (35)	2013	RCT	13 (13)	11 (11)	PAH WHO FC I–IV	55.3 ± 8.7	53.4 ± 12.4	Exercise training	10 weeks in hospital
Ley et al. (9)	2013	RCT	10 (6)	10 (8)	PAH, CTEPH WHO FC II–III	54 ± 14	47 ± 8	Exercise training + RT + mental training	3 weeks in hospital
Mereles et al. (59)	2006	RCT	15 (10)	15 (10)	PAH, CTEPH WHO FC II–IV	47 ± 12	53 ± 14	Exercise training + RT + mental training	3 weeks in hospital 12 weeks at home
Bussotti et al. (67)	2017	Observational study	–	15 (13)	PAH WHO FC II-III	–	45 ± 13	Exercise training + resistance exercises + RT + psychological intervention	4 weeks in outpatient
Becker-Grünig et al. (54)	2013	Prospective cohort	–	20 (16)	CHD-PAH WHO FC II–III	–	48 ± 11	Exercise training + RT + mental training	3 weeks in hospital 12 weeks at home
Chan et al. (72)	2013	Prospective cohort	13 (13)	10 (10)	PAH WHO FC II–III	56 ± 9	53 ± 13	Exercise training	10 weeks in a rehabilitation center
Grünig et al. (AR&T) (70)	2012	Prospective cohort	–	21 (20)	CTD-PAH WHO FC II–IV	–	52 ± 18	Exercise training + RT + mental training	3 weeks in hospital 12 weeks at home
Grünig et al. (ERJ) (60)	2012	Prospective cohort	–	183 (126)	PAH, CTEPH, Others WHO FC II–III	–	53 ± 15	Exercise training + RT + mental training	3 weeks in hospital 12 weeks at home
Nagel et al. (28)	2012	Prospective cohort	–	35 (16)	CTEPH WHO FC II–IV	–	61 ± 15	Exercise training + RT + mental training	3 weeks in hospital 12 weeks at home
Grünig et al. (R) (58)	2012	Prospective cohort	–	58 (42)	PAH, CTEPH, COPD-PH, ILD-PH, Others WHO FC II–IV	–	51 ± 12	Exercise training + RT + mental training	3 weeks in hospital 12 weeks at home
Fox et al. (56)	2011	Prospective cohort	11 (10)	11 (5)	PAH, CTEPH NYHA II–III	57 ± 4	46 ± 5	Exercise training + resistance training	12 weeks in a rehabilitation center
Martínez-Quintana et al. (27)	2010	Prospective cohort	4 (1)	4 (2)	CHD-PAH NYHA II–IV	33 ± 6	23 ± 7	Exercise training + resistance training + educational lesson	12 weeks in a rehabilitation unit
Mainguy et al. (11)	2010	Prospective cohort	–	5 (4)	IPAH WHO FC II–III	–	40 ± 15	Exercise training + single muscles training	12 weeks in a rehabilitation center

AR&T, Arthritis Research & Therapy; CBT, cognitive behavioural therapy; CHD, congenital heart disease; COPD, chronic obstructive pulmonary disease; CTD, connective tissue disease; CTEPH, chronic thromboembolic pulmonary hypertension; ERJ, European Respiratory Journal; ILD, interstitial lung disease; IPAH, idiopathic pulmonary arterial hypertension; NYHA, New York Heart Association Class; OMT, osteopathic manipulative treatment; PAH, pulmonary arterial hypertension; PH, pulmonary hypertension; RCT, randomized control trial; RT, respiratory training; WHO FC, World Health Organization Functional Class.

### 3.1 Exercise training

Restricted exercise capacity is a hallmark symptom of PH, profoundly impacting patients’ quality of life. During physical activity, the body relies on the integrated functions of the respiratory and cardiovascular systems to meet the increased metabolic demands of skeletal muscle contraction. In individuals with PH, impairments in both systems significantly limit their exercise tolerance. Beyond central cardiopulmonary dysfunction, emerging evidence has highlighted the contribution of peripheral abnormalities to exercise intolerance in PH. These include a reduced number and density of capillaries in skeletal muscle, diminished oxidative enzyme activity, and muscle fiber atrophy—all of which negatively influence muscle oxygen utilization and endurance capacity (23). Additionally, morphological and functional alterations in peripheral muscle are frequently observed, such as decreased muscle strength, a lower proportion of type I muscle fibers, and a metabolic shift favoring anaerobic pathways over aerobic metabolism (24). These changes collectively exacerbate exercise limitation and reduce quality of life.

Exercise training, as an economical and easily scalable health intervention, has received substantial support from evidence-based medical research for its multiple health benefits. Physiologically, exercise training significantly enhances cardiopulmonary function, optimizes body composition, improves insulin sensitivity, and regulates lipid metabolism. Psychosocially, its effects are equally remarkable, including alleviating depressive and anxious symptoms, enhancing cognitive function, and delaying the aging process. This multi-dimensional health promotion effect is particularly important for patients with chronic diseases, providing a practical and non-pharmacological intervention option for their disease management and health improvement. It has the potential not only to enhance functional capacity but also to mitigate disease progression by reversing some of the underlying pathophysiological mechanisms. This dual effect—both therapeutic and prognostic—renders exercise training a compelling adjunct in disease management (25–28). Once, there were concerns that increased shear stress on the pulmonary vasculature during exercise might accelerate vascular remodeling and worsen PH. Consequently, exercise training was not routinely recommended. However, recent studies have demonstrated that, when properly supervised, exercise training is safe and beneficial for individuals with cardiopulmonary diseases, including PH (29). Improvements have been noted in cardiorespiratory function, physical performance, and overall quality of life.

Patients with PH constitute a particularly vulnerable population, exhibiting degrees of functional impairment and poor long-term prognosis comparable to, or even exceeding, those observed in heart failure (30–32). These outcomes are primarily driven by progressive increases in pulmonary vascular resistance, declining right ventricular function, and systemic involvement affecting multiple organ systems. The 2022 European Society of Cardiology (ESC) Guidelines for the diagnosis and treatment of PH now advocate for structured rehabilitation therapy in stable PH patients. Specifically, exercise training should be undertaken under professional supervision and in combination with optimized pharmacotherapy. This reflects a paradigm shift, positioning exercise as a safe and effective adjunctive intervention in well-compensated PH patients. In clinical settings, exercise training typically begins under hospital supervision, with the initial intensity set at 60–80% of peak oxygen uptake (peak VO₂) or 40–60% of maximal heart rate (29). It is critical to initiate exercise training at low intensity, progressively increasing workload based on individual tolerance. Continuous monitoring of heart rate (<120 beats/min) and oxygen saturation (>85%) is essential to ensure safety during exercise training sessions (29). Given constraints such as cost and hospitalization duration, continuous inpatient rehabilitation therapy may not be feasible for all patients. Therefore, structured outpatient or home-based exercise training programs are encouraged during the maintenance phase. Approved pharmacological therapies reduce right ventricular afterload and enhance cardiac output, while exercise training addresses peripheral dysfunction—together synergistically improving oxygen delivery and utilization.

Evidence supports the efficacy of supervised home-based rehabilitation therapy in PH, demonstrating improvements in exercise capacity, lower limb muscle strength, activities of daily living (ADL), and health-related quality of life (33). Aerobic training, particularly low-to-moderate intensity, is the modality of choice, whereas high-intensity isometric exercises are generally discouraged due to the risk of syncope. Notably, aerobic exercise may also improve sleep quality and reduce fatigue degree, further contributing to overall patient well-being (34, 35). Additionally, exercise training significantly improved vascular stiffness and right ventricle function, and there is growing interest in the anti-inflammatory effects of exercise on markers including C-Reactive Protein (CRP) and Tumor Necrosis Factor-alpha (TNF-*α*) as a potential therapeutic mechanism in PH (36, 37). Inflammatory factors play a significant role in the development of PH by driving persistent inflammation and promoting pulmonary vascular remodeling. Reducing inflammatory responses can alleviate the progression of PH. Acute bouts of exercise elevate stress hormone levels, reflecting an immediate stress response. In contrast, long-term exercise training appears to attenuate this response, potentially improving the body’s ability to manage chronic stress and systemic inflammation (38). In summary, exercise training represents a valuable and evidence-based strategy for improving functional status, quality of life, and possibly disease trajectory in patients with PH. When implemented appropriately and in conjunction with targeted medical therapy, it can significantly contribute to comprehensive disease management. However, it must be strictly noted that patients with PH who have contraindications must not undergo exercise training (12). Different types of PH require tailored rehabilitation therapy to improve cardiopulmonary function and enhance muscle strength. Additionally, for patients with PH secondary to left heart disease or lung disease, rehabilitation therapy should primarily focus on controlling the underlying primary disease.

### 3.2 Inspiratory muscle training

Although exercise intolerance in patients with PH has long been mainly attributed to cardiopulmonary hemodynamic disorders caused by increased pulmonary vascular resistance and increased right ventricular afterload, such as limited right ventricular output, insufficient cardiac output compensation caused by increased PH, ventilation-perfusion imbalance and hypoxemia. Peripheral muscle dysfunction is usually a direct consequence of hemodynamic problems in the PH center and studies have shown that the degree of impairment of respiratory muscle function in PH is greater than that of the surrounding skeletal muscle strength (39, 40). The impaired systemic oxygen uptake rate, as a direct physiological link between central circulation defects and peripheral metabolic dysfunction, forms a harmful feedback loop. Central problems deteriorate peripheral function, and the deterioration of peripheral function further limits overall motor ability and quality of life. This complex interaction indicates that to achieve effective treatment of PH, a multi-pronged strategy must be adopted to address both central hemodynamic abnormalities and peripheral muscle pathological issues simultaneously.

In recent years, accumulating evidence now highlights the significant role of peripheral factors—particularly skeletal and respiratory muscle impairments. These peripheral changes often precede measurable hemodynamic deterioration, further limiting exercise capacity even in early stages of disease. Respiratory muscle dysfunction, especially involving the diaphragm, has emerged as a critical contributor to ventilatory inefficiency and exercise limitation in PH. During exercise training, compromised respiratory muscle function impairs oxygen delivery, predisposing patients to hypoxemia. Diaphragmatic muscle biopsies from patients with PH reveal pathological changes such as muscle fiber atrophy, reduced contractile force, and decreased capillary density (41, 42). These structural alterations culminate in weakened diaphragmatic performance, which contributes to increased dyspnea, reduced ventilatory efficiency, and maladaptive respiratory patterns. Intervention measures should not only be limited to enhancing cardiopulmonary capacity, but also focus on the training of peripheral muscles, especially respiratory muscles. Currently, the main rehabilitation therapy for patients with PH involves training of the inspiratory muscles.

Inspiratory muscle training (IMT) has gained attention as a non-pharmacological intervention to enhance respiratory mechanics and overall functional capacity in PH. IMT strategies encompass abdominal breathing, pursed lip breathing, inspiratory resistance training using threshold devices, and dynamic exercise protocols combined with respiratory control techniques. Abdominal breathing is a breathing technique characterized by the primary use of the diaphragm muscle to draw air deeply into the lungs, resulting in visible expansion of the abdomen rather than the chest. Pursed lip breathing is a controlled breathing technique where you inhale slowly through the nose and exhale gently through pursed lips, as if blowing out a candle or whistling softly. The primary goal is to prolong exhalation and create back-pressure in the airways. A study conducted the first prospective clinical trial of IMT in patients with PH (43). The results show that IMT can serve as an important treatment option to help PH patients improve their exercise capacity and quality of life, and reduce fatigue and breathing difficulties. External diaphragmatic pacemakers (EDP) can enhance the elasticity of the diaphragm and alleviate diaphragmatic atrophy, thereby improving the respiratory function of patients. EDP, as a non-invasive rehabilitation method, can be combined with IMT and applied to patients with PAH. These interventions aim to strengthen respiratory musculature, reduce the sensation of breathlessness, and improve exercise tolerance and quality of life in patients with PH (44). In summary, a comprehensive understanding of the hemodynamic burden and respiratory muscle dysfunction in patients with PH underscores the importance of implementing integrated, multidisciplinary rehabilitation therapy. The combination of various rehabilitation approaches may offer a feasible and clinically valuable adjunctive therapy to improve functional status and quality of life in this complex patient population.

### 3.3 Neuromuscular electrical stimulation

With the advancement of research, it has become evident that exercise training can exert beneficial effects in patients with PH. However, due to the progressive nature of the disease and its associated symptoms, not all patients are suitable candidates for participation in structured exercise training. Individuals with overt signs or symptoms of advanced right-heart failure, including chest pain, dyspnea, cyanosis of the lips or skin, profound fatigue, near-syncope, abdominal distension, peripheral edema, or hypotension, must engage in exercise training only with the utmost caution and under CPT supervision (45). Neuromuscular Electrical Stimulation (NMES) is a therapeutic technique that employs low-frequency pulsed electrical currents to stimulate nerves and elicit muscle contractions, thereby enhancing muscle function. As a non-invasive and adjustable modality, NMES is widely utilized to improve muscle strength, increase joint range of motion, reduce edema and muscle atrophy, promote tissue healing, and alleviate pain (46, 47). Compared with traditional exercise training, NMES demonstrates significant advantages in situations where movement is not possible or during the early stages of rehabilitation therapy. In patients with chronic obstructive pulmonary disease (COPD), studies have found that NMES can significantly enhance the quality of the quadriceps femoris and effectively improve its function. This is of great significance for the improvement of the overall physical function of patients, especially for those in the stage of severe COPD (48). This result provides support and basis for applying NMES as an innovative treatment approach to patient groups who are unable to undergo conventional pulmonary rehabilitation program for various reasons. There are also similar studies on PH, but the current evidence is limited. Could we consider using NMES as an auxiliary means for routine exercise training and as an alternative method for patients who cannot tolerate physical activity due to the advanced severity of the disease? Notably, NMES imposes a lower metabolic load on the cardiopulmonary system, making it a more tolerable and potentially safer intervention for patients with significant exercise limitations. By improving peripheral muscle strength and functional mobility, NMES represents a valuable therapeutic option in the comprehensive management of PH.

### 3.4 Psychological interventions

Previous studies on PH mainly focused on reducing the mortality and morbidity rates of patients. However, in recent years, the research perspective has gradually shifted to the profound impact of this disease on patients’ activity ability and life participation. Affected by symptoms such as breathing difficulties and fatigue, PH patients often have difficulty completing some household chores, and in severe cases, they even cannot have long conversations or walk. The limitation of activities leads many patients to have to give up the activities they were once passionate about, and their social scope significantly shrinks. Some patients further actively reduce interaction with relatives and friends due to the concern that “others do not understand.” This widespread functional limitation and participation restriction is inevitably accompanied by significant psychological and social burdens. When with the help of CPT, IMT can be implemented more safely and effectively, leading to greater improvements in respiratory muscle strength, exercise capacity, and dyspnea in symptomatic PH patients (49). PT and OT design and implement individualized functional training programs, aiming to directly improve patients’ activity ability and the efficiency of daily task execution. This improvement in functional status not only helps to alleviate the frustration caused by physiological limitations but also effectively relieves the resulting psychological stress, enhancing the patients’ coping ability and self-efficacy. Ultimately, comprehensive rehabilitation therapy is expected to significantly improve the overall quality of life of this vulnerable group.

## 4 The role of rehabilitation in pulmonary hypertension and its potential adverse effects

In the assessment of rehabilitation outcomes for patients with PH, various indicators such as 6-minute walk test, Borg Dyspnea Scale, arterial blood gas variables, New York Heart Association(NYHA) function class, pulmonary function tests, echocardiogram, as well as Quality-of-Life Scores (QoL scores) and the number of annual acute exacerbations are typically combined to comprehensively reflect exercise endurance, symptom improvement, optimization of cardiopulmonary function, and long-term prognosis. This review mainly presents 6-minute walk test, maximal oxygen uptake and peak VO₂, and quality of life.

### 4.1 Exercise capacity

#### 4.1.1 Six-minute walk test

In recent years, invasive cardiopulmonary exercise testing, performed alongside right heart catheterization, has garnered significant attention as a valuable tool for accurately identifying the underlying cardiopulmonary mechanisms driving exercise intolerance in complex clinical conditions (50). However, invasive procedures carry various risks, are costly, have high technical requirements, and may also have the problem of poor patient tolerance. In parallel, the 6-Minute Walk Test (6MWT) remains a simple, cost-effective, safe, and widely accepted method for assessing functional exercise capacity. Developed initially to evaluate endurance in adults with chronic heart failure (51), the 6MWT measures the maximum distance a patient can walk at a self-paced speed on a flat, hard surface during 6 min, allowing for rest as needed, reflecting submaximal exercise capacity. This test provides a practical indicator of physical function and quality of life, as it reflects the ability to perform everyday activities (52). However, performance on the 6MWT is influenced by multiple factors, including gender, age, height, weight, comorbidities, and oxygen requirements.

Evidence-based research supports the utility of the 6MWT in evaluating exercise capacity, monitoring the efficacy of medical interventions, and providing prognostic insights for patients with cardiovascular or respiratory diseases. Its applications have broadened in recent years, particularly as rehabilitation therapy have gained traction. The 6MWT has proven to be an effective tool in assessing treatment impact on exercise capacity and has frequently served as a primary endpoint in trials involving patients with PH (53–56). Notably, predictive equations have been developed to estimate peak VO₂ from 6-minute walk distance (6MWD), offering an alternative to cardiopulmonary exercise testing for patients unable to undergo more rigorous assessments (57).

Clinical trials underscore the benefits of exercise training in patients with PH. In prospective studies, significant improvements in prognostic parameters—including 6MWD, peak VO₂, exercise capacity, and quality of life—have been observed, along with upgrades in WHO functional class (WHO FC). These benefits were most pronounced in patients initially classified as WHO FC IV compared to WHO FC II/III (58). In the randomized controlled trial (RCT) involving patients with severe chronic PH, a 15-week exercise training led to significant gains in 6MWD as compared to the control group (59). Similarly, in patients with PH and chronic thromboembolic pulmonary hypertension (CTEPH), a rehabilitation therapy combining inpatient and home-based interventions—featuring low-intensity aerobic training and respiratory therapy—yielded clinically meaningful improvements in exercise tolerance and 6MWD (60, 61).

Further supporting these findings, a 6-month regimen of combined aerobic and strength training demonstrated substantial improvements in 6MWD without exercise-related adverse events, with benefits persisting for at least 3 months post-intervention (62). Long-term exercise training have proven to be safe and effective in enhancing functional capacity, psychological well-being, and quality of life in patients with PH. Targeted strengthening of lower extremity muscles, particularly the quadriceps, has shown to significantly improve walking ability in patients with muscle atrophy or weakness, as increased muscle strength directly correlates with greater 6MWD (7, 63). Additionally, psychological factors play a critical role in 6MWT performance. Interventions such as psychological support, motivational enhancement, and behavioral therapies have been shown to boost patient confidence and adherence to rehabilitation programs, indirectly improving outcomes on the 6MWT.

A study of a home-based rehabilitation therapy for PH patients further highlights the efficacy of exercise training in improving both functional parameters and quality of life. Remarkably, patients maintained significant gains in respiratory muscle strength and 6MWD even 6 months after completing the rehabilitation therapy (64). These findings collectively underscore the significant and sustained benefits that exercise training, as an integral component of a comprehensive pulmonary rehabilitation program, can deliver for the physical and psychological well-being of patients with PH. This reinforces the critical importance of incorporating such evidence-based rehabilitation therapy into the standard comprehensive care pathway.

#### 4.1.2 Maximal oxygen uptake and peak oxygen uptake

Maximal oxygen uptake (VO₂ max) refers to the highest amount of oxygen an individual can intake and utilize per unit time, serving as a key indicator of cardiorespiratory endurance. An increase in VO₂ max reflects improved cardiorespiratory fitness, and previous studies have demonstrated that VO₂ max in patients with PH significantly improves following exercise training (65). Similarly, peak VO₂ is an essential measure of aerobic capacity and cardiorespiratory function, representing the maximum ability of the body to uptake, deliver, and utilize oxygen during exercise. Evidence suggests that mean peak VO₂ also increases significantly after exercise training (60, 66).

### 4.2 Quality of life

For patients with PH, the physiological functional impairments caused by the disease progression will significantly reduce their quality of life. This progressive functional limitation not only affects the patients’ ability to take care of themselves in daily life, but also leads to a significant decrease in their working ability and social participation. Therefore, in the clinical management of PH, in addition to the treatment targeting the disease itself, special attention needs to be paid to the assessment and improvement of the patients’ quality of life. Rehabilitation therapy is part of the recommended management plan of PH and is important to better quality of life and exercise tolerance. Exercise training, as an adjunct to disease-targeted pharmacotherapy, can significantly enhance the quality of life in patients with PH (59, 66–69). The study have found that the patients with connective tissue disease-associated pulmonary hypertension (CTD-PH) were given exercise training on the basis of drug treatment, and their quality of life scores were significantly improved after exercise training (70). Compared to control groups, IMT has been shown to improve brachial blood pressure and central blood pressure, alleviate dyspnea, increase respiratory muscle strength, enhance pulmonary carbon monoxide diffusion capacity at total lung capacity, and strengthen knee extensor muscles. Additionally, IMT improves exercise capacity, physical activity, and ADL, while reducing fatigue and anxiety, ultimately enhancing overall quality of life (71). The 36-Item Short Form Health Survey (SF-36), which comprises two composite scales, demonstrates high validity, reliability, and stability in PH. The two summary scores assess physical health and mental health. Higher scores on each subscale indicate better health status.

Studies have shown that long-term exercise training leads to significant improvements in quality of life in PH patients, as measured by the SF-36 (62), as well as through rehabilitation therapy such as plate exercise training (54, 72). Following the rehabilitation therapy, participants in the exercise group demonstrated significant improvements in QoL scores, particularly in mental health, along with positive trends in physical functioning and socialization compared to the control group (60, 73). Collectively, these findings suggest that scientifically structured rehabilitation therapy plays a crucial role in improving both the physical and mental health, as well as the overall quality of life, of patients with PH.

### 4.3 Adverse events during the intervention

A number of high-quality studies have confirmed that during the individualized rehabilitation therapy for patients with PH under the guidance of CPT, the incidence of adverse events is extremely low. Patients with PH generally do not experience significant adverse effects during rehabilitation interventions. Vigorous aerobic exercise training was not associated with substantial declines in left ventricular systolic or diastolic function in women with PH. Aerobic exercise training may be beneficial for reducing afterload and may preserve left ventricular diastolic function (74). However, some reports indicate that despite the notable improvements in exercise tolerance and quality of life achieved through rehabilitation therapy, there remains a risk of adverse events due to the unique cardiopulmonary limitations and reduced exercise tolerance characteristic of PH patients. The most frequently reported adverse events are cardiac arrhythmias, dyspnea, and respiratory infections (55, 60, 75). For example, in one study involving 183 PH patients who underwent 3 weeks of hospital-based exercise training followed by a home-based program, adverse events such as respiratory infections and syncope were observed in 13% of participants. These findings suggest that while exercise training is an effective adjunct therapy for PH patients, it is not entirely without risk and warrants careful monitoring throughout the intervention period (58).

## 5 Future directions and opportunities for rehabilitation in pulmonary hypertension

In recent years, the integration of Virtual Reality (VR) technology into rehabilitation therapy has garnered increasing attention, particularly in the management of chronic diseases, where it has demonstrated unique potential. VR seamlessly merges real and virtual environments, enabling the replication of various scenarios to create immersive experiences for users (76). Patients with PH often experience chest discomfort, and invasive procedures such as right heart catheterization or injections may further contribute to pain and distress. Studies have shown that VR technology can effectively distract patients from procedural pain by providing highly immersive virtual environments, thereby significantly reducing their perception of treatment-related discomfort. As an emerging adjunctive therapy, VR offers the advantage of alleviating pain through non-pharmacological means, thus avoiding the potential side effects associated with analgesic medications.

Moreover, PH patients frequently bear significant psychological burdens, including anxiety, depression, and other emotional disturbances, particularly as the disease progresses, all of which directly affects their quality of life. To effectively improve the quality of life of these patients, comprehensive rehabilitation therapy is necessary. On the one hand, VR can provide relaxing experiences—such as virtual exposure to natural environments and guided meditation—that help reduce emotional stress and psychological distress. On the other hand, VR includes low-intensity exercise training to improve cardiorespiratory fitness, thereby continuously improving the quality of life of patients. VR technology facilitates patient engagement in such exercises within a virtual environment, including simulated walking, cycling, and other activities. Real-time monitoring of physiological parameters, such as heart rate and oxygen saturation, through sensors ensures that exercise intensity remains within safe limits. The gamification and interactive features of VR further enhance the appeal of rehabilitation therapy, mitigating monotony and boosting patient participation and motivation.

In addition to its therapeutic applications, VR holds significant promise in medical education. Three-dimensional visualization allows VR to vividly illustrate the pathophysiology and treatment processes of PH, aiding patients in comprehending disease progression and drug mechanisms. Simulations of medication use and standardized rehabilitation instructions can strengthen patients’ adherence to treatment and foster a sense of empowerment and confidence. Despite its considerable promise, the widespread adoption of VR technology remains limited by the high costs associated with equipment and software. Furthermore, individual variability means that not all patients tolerate VR well. Some patients may experience dizziness, nausea, imbalance, or coordination difficulties, leading to poor adaptability (77). Significantly, although the potential of VR in PH management is substantial, current research in this area is limited. Further RCT are urgently needed to validate its efficacy and safety, thereby establishing a stronger clinical evidence base for the application of VR technology in the treatment of PH.

## 6 Conclusion

In the management of PH, pharmacological therapy remains the cornerstone, as medications can improve patients’ hemodynamics. However, rehabilitation is emerging as an effective adjunct therapy that is garnering increasing attention. Systematic rehabilitation has been shown to significantly enhance exercise tolerance and quality of life in PH patients. It is important to emphasize that rehabilitation is not a replacement for medication, but rather serves as a vital complementary approach. Patients should receive optimized pharmacological treatment for at least 3 months before initiating a structured rehabilitation therapy to ensure clinical stability. By integrating systematic rehabilitation with optimized medication, a synergistic effect can be achieved, resulting in improved quality of life and functional status for PH patients. This comprehensive management strategy embodies the principle of patient-centered care, focusing not only on effective disease control but also on overall health, long-term prognosis, and quality of life, striving for an optimal balance between survival and well-being.

**Figure 1:**
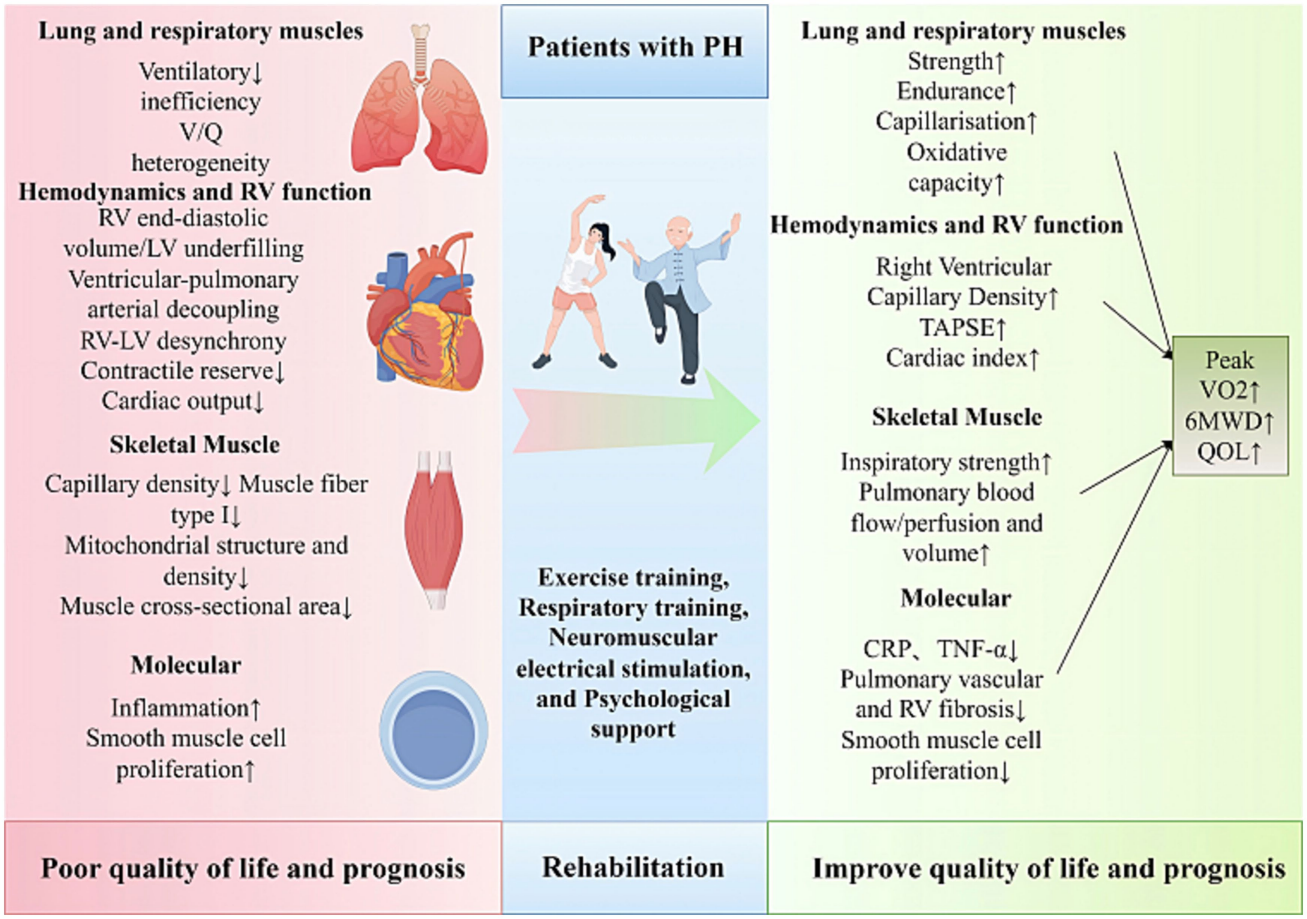
Clinical benefits of exercise training for pulmonary hypertension. 6MWD, 6-Minute Walk Distance; CRP, C-Reactive Protein; LV, Left Ventricle; QOL, Quality of Life; RV, Right Ventricle; TNF-*α*, Tumor Necrosis Factor-alpha; TAPSE, Tricuspid Annular Plane Systolic Excursion; V/ Q, Ventilation/ Perfusion.

A variety of rehabilitation therapy approaches, including exercise training, IMT, NMES, and psychological interventions, have demonstrated benefits in enhancing exercise capacity and cardiorespiratory function in PH patients. Gradual increases in exercise intensity can improve the body’s oxygen utilization efficiency without placing undue strain on the heart. Exercise training during rehabilitation therapy also promotes the release of endorphins, improves mood, and enhances cognitive performance. As a result, patients’ functional abilities and ADL are enhanced to varying degrees. Importantly, the safety and feasibility of rehabilitation in PH have been increasingly validated, with studies indicating that rehabilitation is safe when conducted under professional supervision. In addition, patient and family education is a critical component of rehabilitation, fostering a better understanding of the disease and the importance of ongoing rehabilitation, thereby enhancing self-management capabilities (Figure 1).

Looking forward, more high-quality RCTs are needed to clarify the specific indications and applicability of rehabilitation therapy across different PH patient subtypes. Further investigation is warranted to establish evidence-based exercise prescriptions for PH, including optimal modalities, intensity parameters, intervention duration, supervision requirements, and implementation settings. Long-term follow-up studies are essential to evaluate the sustainability of rehabilitation benefits and to assess their impact on key outcomes such as time to clinical deterioration and survival. Furthermore, comprehensive clinical data regarding adverse events during rehabilitation therapy are necessary to inform the continual refinement of rehabilitation programs for PH patients.
